# Evolution of Oxidative Phosphorylation (OXPHOS) Genes Reflecting the Evolutionary and Life Histories of Fig Wasps (Hymenoptera, Chalcidoidea)

**DOI:** 10.3390/genes11111353

**Published:** 2020-11-15

**Authors:** Yi Zhou, Dawei Huang, Zhaozhe Xin, Jinhua Xiao

**Affiliations:** Institute of Entomology, College of Life Sciences, Nankai University, Tianjin 300071, China; 1120170366@mail.nankai.edu.cn (Y.Z.); huangdw@nankai.edu.cn (D.H.); 1120180392@mail.nankai.edu.cn (Z.X.)

**Keywords:** oxidative phosphorylation, positive selection, fig wasps

## Abstract

Fig wasps are a peculiar group of insects which, for millions of years, have inhabited the enclosed syconia of fig trees. Considering the relatively closed and dark environment of fig syconia, we hypothesize that the fig wasps’ oxidative phosphorylation (OXPHOS) pathway, which is the main oxygen consumption and adenosine triphosphate (ATP) production system, may have adaptively evolved. In this study, we manually annotated the OXPHOS genes of 11 species of fig wasps, and compared the evolutionary patterns of OXPHOS genes for six pollinators and five non-pollinators. Thirteen mitochondrial protein-coding genes and 30 nuclear-coding single-copy orthologous genes were used to analyze the amino acid substitution rate and natural selection. The results showed high amino acid substitution rates of both mitochondrial and nuclear OXPHOS genes in fig wasps, implying the co-evolution of mitochondrial and nuclear genes. Our results further revealed that the OXPHOS-related genes evolved significantly faster in pollinators than in non-pollinators, and five genes had significant positive selection signals in the pollinator lineage, indicating that OXPHOS genes play an important role in the adaptation of pollinators. This study can help us understand the relationship between gene evolution and environmental adaptation.

## 1. Introduction

The symbiotic relationship between fig trees (Moraceae, *Ficus*) and their pollinating fig wasps (or abbreviated as pollinators) (Chalcidoidea, Agaonidae) can be traced back to 75 million years ago and represents extreme obligate mutualism [1]. Fig trees have enclosed inflorescences (syconia), which have a narrow opening (ostiole) connecting the inside and outside world. Therefore, the internal environment of fig syconia should be closed and lightless, and the oxygen content in the fig syconia may also be different from that of the outside atmosphere. The figs also provide habitats for other wasps (Hymenoptera, Chalcidoidea), which do not pollinate figs (that is, non-pollinators). Pollinators and non-pollinators have different lifestyles related to figs, as a mature female pollinator leaves the natal fig syconium to enter a new receptive syconium for pollination and oviposition, while non-pollinators do not pollinate, and most of them only lay eggs outside syconia through the fig wall to the fig ovaries [2]. For pollinators, the process of drilling into the fig syconia is not easy, and they have flat heads and a row of teeth of mandibular appendages, which can help in pushing bracts of the ostiole [3]. Furthermore, previous studies have shown that the pollinators, which emerged during the Eocene, are older than non-pollinators, which emerged during the Oligocene or Miocene [4]. Therefore, the fig pollinators and non-pollinators display different life and evolutionary histories related to the peculiar living environment within fig syconia, implying that they may have different evolutionary patterns in terms of the oxygen utilization strategy and energy consumption.

The oxidative phosphorylation (OXPHOS) pathway is the main oxygen consumption and adenosine triphosphate (ATP) production system in eukaryotic cells [5]. It is composed of five complexes. Except for complex II, in which the proteins are all encoded by nuclear genes, the remaining four complexes (complex I, III, IV, and V) are composed of subunits encoded by both mitochondrial and nuclear genes [6]. Therefore, maintenance of the function of OXPHOS may involve the co-evolution of mitochondrial genes and nuclear genes. Generally, mitochondrial genes evolve faster than most nuclear genes, so nuclear genes related to OXPHOS may also show faster evolution rates than the nuclear genes which are not involved in OXPHOS [7,8]. Many studies have been carried out to detect the evolutionary patterns of the OXPHOS system in other insects, such as fruit flies [9]; parasitic wasps *Nasonia* [10]; and a comparison of Hymenoptera, Lepidoptera, Coleoptera, and Diptera [11]. The articles on the OXPHOS genes of fig wasps are mostly limited to mitochondrial-coding genes [12,13], and there is only one work on mitochondrial and nuclear OXPHOS genes based on unpublished data from our laboratory [14]. In fact, considering the special living environment of fig wasps related to figs, the evolution of genes related to the OXPHOS pathway is a topic worth exploring.

In this study, we used mitochondrial and nuclear genomic data to explore the evolutionary pattern of OXPHOS-related genes in fig wasps, in order to detect whether OXPHOS genes display different evolutionary patterns in the adaptation of pollinators and non-pollinators to living within fig syconia.

## 2. Materials and Methods 

### 2.1. Sequence Determination

We used mitochondrial genomes, nuclear genomes, and transcriptome data from 11 fig wasps, including six pollinators and five non-pollinators (Table S1). The species classification was provided by the website (http://www.figweb.org/Fig_wasps/Classification/index.htm).

The nuclear-encoded OXPHOS genes for *Drosophila melanogaster* (dme00190), *Nasonia vitripennis* (nvi00190), and *Apis mellifera* (ame00190) were downloaded from the KEGG database [15]. We performed TBLASTN searches of the whole genome sequences of fig wasps using the amino acid sequences of *D. melanogaster*, *N. vitripennis*, and *A. mellifera* as queries. If the BLAST E-scores were lower than 10^−4^, the sequences were mapped to the genome to identify the positions of each exon and modified with the actual transcripts shown in the transcriptomic data, using IGV v2.4.14 [16,17]. If the quality of transcriptome data was poor and there were no clear exons on IGV, we used the Softberry website (www.softberry.com/berry.phtml?topic=fgenes_plus&group=programs&subgroup=gfs) to predict the complete coding sequences of genes. Then, the genes were extracted from genomes and translated to protein sequences using BioEdit v7.0.9.0. Using these protein sequences as queries, we searched for the conserved domains on NCBI CD-search tool [18]. 

The complete mitogenomes were annotated using the MITOS webserver [19] and NCBI ORF Finder (https://www.ncbi.nlm.nih.gov/orffinder). When the annotation results from both webservers were different, we compared them with the 13 protein-coding genes of the closely related species *N. vitripennis* (EU746609.1 and EU746613.1) to evaluate the final length of the reading frames. 

In order to obtain a nuclear non-OXPHOS gene set, we used OrthoMCL v2.0.9 [20] to screen the single-copy orthologous genes of 11 fig wasp species (the genome accessions in Table S1). Then, the OXPHOS genes were removed from the single-copy gene set. Finally, a set of 4622 single-copy non-OXPHOS genes was obtained.

### 2.2. Phylogenetic Reconstruction

**Figure 1:**
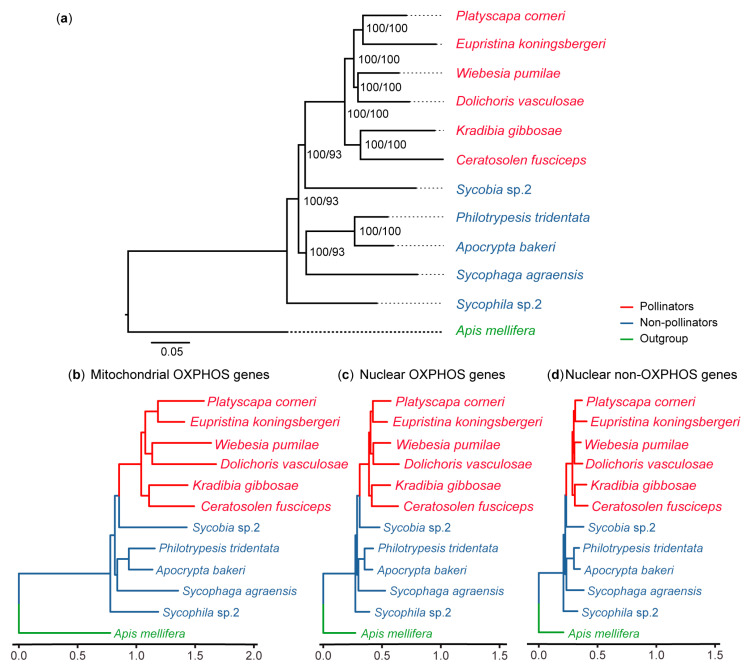
The phylogenetic trees used in this study. Red species represent pollinators, blue species represent non-pollinators, and green species represent the outgroup. (**a**) Phylogenetic tree based on mitochondrial-coding genes and nuclear single-copy orthologous genes. Number on the node represents SH-aLRT%/UFBoot%. Phylogenetic trees based on concatenated amino acid alignments of (**b**) mitochondrial OXPHOS genes, (**c**) nuclear OXPHOS genes, and (**d**) nuclear non-OXPHOS genes. Branch lengths represent the average number of amino acid substitutions per site.

The phylogenetic tree was built based on nuclear single-copy orthologous genes and 13 mitochondrial protein-coding genes. OrthoMCL v2.0.9 [20] was used to identify single-copy orthologous genes in the genomes of 11 fig wasps and *A. mellifera* (GCF_000002195). The amino acids of each gene were aligned using MAFFT v7.313 [21] implemented by PhyloSuite v1.1.16 [22]. Gblocks 0.91b [23] was used to remove poor alignments. These alignment sequences were concatenated by PhyloSuite v1.1.16 [22]. The phylogenetic tree was reconstructed using IQ-TREE v1.6.1 [24] based on the maximum likelihood method with the best model of JTT+F+I+G4 and 5000 ultrafast bootstrap replicates. *A. mellifera* was used as the outgroup. The tree topology (Figure 1a) was used for subsequent analysis.

### 2.3. Estimation of the Amino Acid Substitution Rate

We used the branch length from the outgroup (*A. mellifera*) to each ingroup as the estimation of the amino acid substitution rate, based on the maximum likelihood trees [11]. 

In the mitochondrial-encoded OXPHOS gene category, the protein sequence of each gene was aligned using MAFFT v7.313 [21] and concatenated using PhyloSuite v1.1.16 [22]. The poor blocks were removed using Gblocks 0.91b [23]. Then, the maximum likelihood tree was reconstructed using IQ-TREE v1.6.1 [24] with the automatically selecting model and the tree topology of Figure 1a. The same procedures were carried out for the nuclear OXOPHS gene category and nuclear non-OXPHOS gene category. These phylogenetic trees were visualized and colored with the R package “ggtree” [25].

For each individual gene in every category, the protein sequence was aligned using MAFFT v7.313 [21]. Then, each gene tree was reconstructed based on the topology from Figure 1a using IQ-TREE v1.6.1 [24] with the parameter automatically selecting the best fitting model.

The branch length from each ingroup species to outgroup species (*A. mellifera*) was retrieved using Newick Utilities v1.6 [26], which was used as the amino acid substitution rate of the gene [11]. The Wilcoxon rank sum test in R software was employed to test the amino acid substitution rate difference between the pollinators and non-pollinators. The pairwise Wilcoxon rank sum test was used to test the difference between mitochondrial OXPHOS, nuclear OXPHOS, and nuclear non-OXPHOS genes. The *p* values for multiple comparisons were adjusted by Holm correction [27].

### 2.4. Natural Selection Analysis

We used codeml implemented in PAML v4.9f [28] to test the natural selection for each gene in mitochondrial and nuclear OXPHOS gene categories. The nonsynonymous (*d*N) to synonymous (*d*S) rate (ω = *d*N/*d*S) ratios represent the changes of selective pressures (ω = 1, neutral evolution; ω < 1, purifying selection; ω > 1, positive selection). The tree topology (Figure 1a) without *A. mellifera* was used for the selection analysis. 

The one-ratio models were used to estimate the single ω ratio for all branches in the phylogenetic tree. The free-ratio models [29,30] were used to obtain an independent ω ratio for each branch. We compared the ω ratio of pollinators and non-pollinators in each gene using the Wilcoxon rank sum test in R software. The boxplots of ω ratios were made using “ggpubr” in the R package.

Positive selection may act in short episodes, affecting only a few sites along particular lineages. The modified branch-site model A [31] and its null model (branch-site model A with ω_2_ = 1 fixed) were used to test for the positive selection of sites along foreground branches (pollinator lineages). The likelihood ratio test was used to test whether the alternative model was significantly better than the null model and Bayes empirical Bayes (BEB) [32] analysis was employed to identify positive sites. When the likelihood ratio test was significant and the posterior probability was greater than 95%, we considered this gene to be a positively selected gene.

## 3. Results

### 3.1. OXPHOS Gene Annotation

We manually annotated OXPHOS genes for 11 fig wasp species by using the genomic and transcriptomic data. A total of 72 genes related to OXPHOS were found, but the copy numbers were different in different species (Table S2). Finally, we found that 30 genes were single-copy orthologs with completed conserved domains for every species, and 13 orthologous genes had multiple copies in some species (Table S2). In particular, the COX4 gene was the most special in that it had a single copy in each pollinator species, but two copies in each non-pollinator (Table S2). The gene tree of COX4 showed that the lineage of one copy (namely COX4.2) in non-pollinators exhibited a basal position and another copy (namely COX4.1) in non-pollinators was clustered with COX4 in pollinators (Figure S1). The 30 single-copy genes were used in subsequent analyses as the gene category of “nuclear OXPHOS genes”.

### 3.2. Amino Acid Substitution Rate Analysis

**Figure 2:**
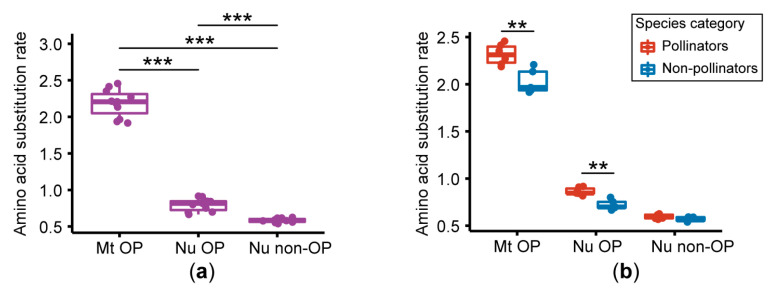
The amino acid substitution rate estimation based on concatenated mitochondrial OXPHOS genes, concatenated nuclear OXPHOS genes, and concatenated nuclear non-OXPHOS genes. **, *p* < 0.01 and ***, *p* < 0.001. Abbreviations: Mt OP, mitochondrial OXPHOS genes; Nu OP, nuclear OXPHOS genes; Nu non-OP, nuclear non-OXPHOS genes. (**a**) The comparisons of the amino acid substitution rate for mitochondrial OXPHOS, nuclear OXPHOS, and nuclear non-OXPHOS genes. The *p* values were determined by the pairwise Wilcoxon rank sum test and adjusted by Holm correction. (**b**) Comparisons of the amino acid substitution rate for pollinators and non-pollinators in three gene categories. The *p* values were determined by the Wilcoxon rank sum test. Red boxplots represent pollinators and blue boxplots represent non-pollinators.

We compared the amino acid substitution rate of mitochondrial-encoded OXPHOS genes, nuclear OXPHOS genes, and nuclear non-OXPHOS genes, based on the maximum likelihood trees (Figure 1b–d). The results of three concatenated sequences showed that the highest substitution rate was found in mitochondrial genes, followed by nuclear OXPHOS genes, and finally nuclear non-OXPHOS genes (Figure 2a and Table S3). 

**Figure 3:**
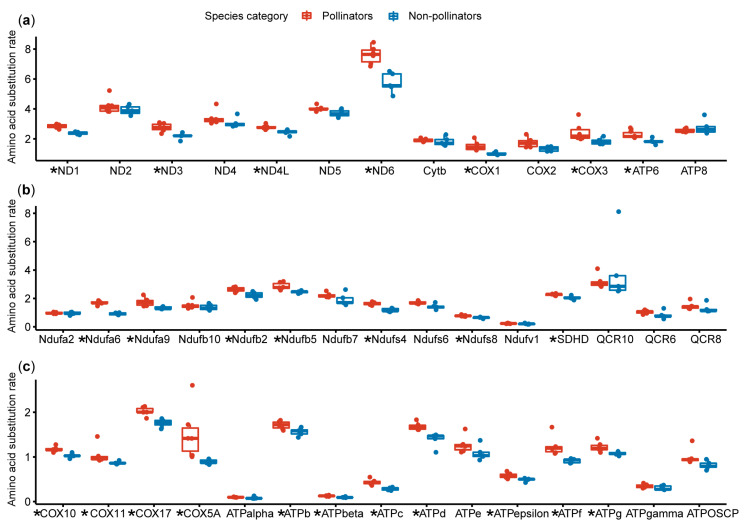
Amino acid substitution rate estimation of each OXPHOS-related gene. A comparison of pollinators and non-pollinators based on 13 mitochondrial genes (**a**); nuclear-encoded genes in OXPHOS complex I, II, and III (**b**); and nuclear-encoded genes in OXPHOS complex IV and V (**c**). Red boxplots represent pollinators and blue boxplots represent non-pollinators. Genes with a significant difference in rates are marked with an asterisk.

We also compared the amino acid substitution rates of the genes for pollinators and non-pollinators, and the results showed that the mitochondrial genes of the pollinators exhibited a higher amino acid substitution rate than those of the non-pollinators (*p* < 0.01); nuclear OXPHOS genes displayed the same difference pattern (*p* < 0.01) (Figure 2b and Table S3). However, differences of nuclear non-OXPHOS genes between pollinators and non-pollinators were not significant (*p* > 0.05) (Figure 2b and Table S3). In order to be more specific about the substitution rate of each gene, we compared the substitution rate of each individual gene for pollinators and non-pollinators, and declared that 25 out of the 43 mitochondrial and nuclear OXPHOS genes showed higher amino acid substitution rates in pollinators than in non-pollinators (*p* < 0.05) (Figure 3 and Table S4).

### 3.3. Natural Selection Analysis on the OXPHOS Genes

**Figure 4:**
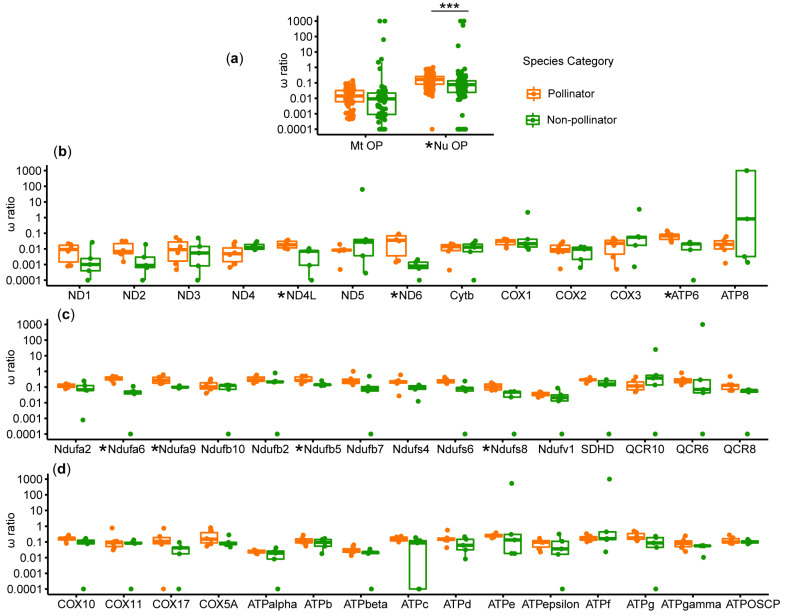
The ω ratios of mitochondrial and nuclear OXPHOS genes. Orange boxplots represent the ω ratio distribution of pollinator species and green boxplots represent the ω ratio distribution of non-pollinators. Genes with a significant difference in ω ratios are marked with an asterisk. ***, *p* < 0.001. Abbreviations: Mt OP, mitochondrial OXPHOS genes; Nu OP, nuclear OXPHOS genes. A comparison of the ω ratios for pollinators and non-pollinators based on (**a**) all mitochondrial and nuclear OXPHOS genes; (**b**) each mitochondrial gene; (**c**) genes involved in nuclear OXPHOS complex I, II, and III; and (**d**) genes related to OXPHOS complex IV and V.

We tested for selective pressure on OXPHOS genes by comparing the ratio of non-synonymous to synonymous substitutions. The one-ratio model results showed that each gene had a ω ratio far smaller than one (Table S5), indicating that all tested genes were under purifying selection. We conducted a comparison of ω ratios for pollinators and non-pollinators on mitochondrial and nuclear OXPHOS genes using the free-ratio model results (Figure 4 and Table S6). The results demonstrated that the median ω ratio of pollinators was higher than that of non-pollinators in nuclear-encoded OXPHOS genes (*p* < 0.05), but there was no significant difference in mitochondrial OXPHOS genes (Figure 4a). At the level of individual genes, pollinators exhibited higher ω ratios than non-pollinators in most genes; however, the differences were only significant in seven genes (Figure 4b–d and Table S6).

Considering that the higher ω ratios of the OXPHOS genes in the pollinators may indicate positive selection or relaxed selection, we used the branch-site model to test for positive selection in pollinators (Table S7). The results showed that five nuclear-encoded OXPHOS genes were under positive selection in the branch of pollinators compared with non-pollinators (Table 1). BEB analysis suggested that eight sites were well-supported (posterior probability > 95%) in the five genes, which encode proteins on complex I (Ndufb5 and Ndufb10), complex IV (COX11), and complex V (ATPd and ATPOSCP), respectively (Table 1).

## 4. Discussion

In this study, we manually annotated the OXPHOS genes in 11 fig wasp species and conducted a thorough molecular evolution analysis, in order to understand whether they present different evolutionary patterns in fig pollinators and non-pollinators, due to their different life history and evolutionary history related to fig syconia. 

Based on the comparative analysis of gene members related to OXPHOS, we found that most orthologous OXPHOS genes are single-copy in fig wasps, but some orthologous genes have multiple copies in several fig wasp species. An interesting result that caught our attention is that COX4, encoding a key subunit of cytochrome c oxidase, has single copies in pollinators and two paralogous copies in non-pollinators. According to the phylogenetic tree of COX4, we can infer that the ancestral species of fig wasps had two copies of COX4, and the fact that the pollinators have only one copy may have been caused by gene loss. There are also two paralogs in vertebrates, except birds [33], and functionally, the genes in mammals are hypoxia-responsive [34], and in fish, they expressed in specific tissues [35]. These previous studies indicate that COX4 genes may play important roles in the adaptive evolution of organisms, enabling them to adapt to different oxygen environments, and our results thus lead us to infer that pollinators and non-pollinators may present different evolutionary patterns in the function of COX4 in their divergent evolutionary histories with figs. Additionally, the proteins encoded by the two paralogs may have been differentiated in non-pollinating fig wasps. 

The level of the amino acid substitution rate can often well-reflect the rate of protein evolution [36]. In this study, the amino acid substitution rate of OXPHOS genes was higher than non-OXPHOS genes, and these results are consistent with previous findings on other insects in Hymenoptera [11]. In view of the rapid evolution of mitochondria in fig wasps [12], the rapid evolution of nuclear-coding OXPHOS may represent compensatory evolution for maintaining function, which supports the viewpoint of the coevolution of mitochondria and the nucleus [7]. This is consistent with previous estimations of OXPHOS evolution patterns among different insect orders [11]. We also found that the concatenated mitochondrial OXPHOS genes and concatenated nuclear OXPHOS genes display higher amino acid substitution rates in pollinators than in non-pollinators, respectively. In the results of individual genes, in 58.1% of mitochondrial and nuclear OXPHOS genes, the amino acid substitution rate of pollinators is significantly higher than that of non-pollinators. This indicates that the evolution of OXPHOS genes in pollinators occurred more rapidly than in non-pollinators.

Genes with a fast evolution rate often have a high number of nucleotide substitutions which are driven by positive selection or neutral mutation [37]. In view of the fact that the OXPHOS-related genes of the pollinator fig wasps may have evolved faster than those of the non-pollinators, we performed an evolutionary selection pressure analysis on these genes. The results of the natural selection analysis based on the branch model in PAML show that the overall ω ratio pattern of pollinators is significantly higher than that of non-pollinators in nuclear OXPHOS genes, suggesting that these genes in pollinators have undergone relaxed purifying selective constraint or positive selection [38]. We used branch-site models and found that five genes are positively selected in pollinators among 43 OXPHOS-related genes. Ndufb5 and Ndufb10 encode accessory subunits, which are not directly involved in catalysis and may be required for stabilizing complex I (NADH: ubiquinone oxidoreductase) [39]. COX11 is a copper-binding protein, and it is essential for complex IV (cytochrome c oxidase) assembly and function [40]. ATPd and ATPOSCP are parts of the peripheral stalk and play vital roles in maintaining the structural stability of complex V (F1Fo ATP synthase) [41,42]. Therefore, complex I, IV, and V of the mitochondrial respiratory chain may play important roles in the adaption of pollinators.

Three genes (Ndufb5, COX11, and ATPd) have significantly higher amino acid substitution rates in pollinators than those in non-pollinators and they are positively selected in the pollinator lineage. Therefore, we speculate that the high substitution rates of these genes have been driven by positive selection. However, there are other genes that are not positively selected, but show high substitution rates, which may be caused by other factors, such as neutral mutations.

Positive selection may drive OXPHOS genes to better adapt to the high energy requirements and hypoxia [43,44,45]. Therefore, we infer that pollinators expend more energy than non-pollinators, which may be needed in the life behaviors of drilling into syconia and pollinating flowers. In the future, we will study the causes of fig–wasp mutualism affecting OXPHOS genes of pollinators and attempt to obtain experimental evidence for positively selected genes affecting the function of OXPHOS.

## 5. Conclusions

In the compact environment of fig syconia, where fig wasps have lived for tens of millions of years, the fig pollinators not only have a longer coevolutionary history with fig trees, but also a longer lifetime inside syconia in their adult life history than the non-pollinators, which may indicate their different evolutionary patterns in the genes of OXPHOS pathway. Our molecular evolution analysis results based on the OXPHOS genes in fig wasps showed that the nuclear-encoded OXPHOS genes had a faster evolutionary rate than the nuclear-encoded non-OXPHOS genes, which may be due to co-evolution with the fast evolution of mitochondrial genes. Our results also found that both mitochondrial and nuclear OXPHOS genes in pollinators were evolving faster than the genes in non-pollinators, which may be driven by positive selection on the pollinators. This study provides us with some evidence on the adaptation of insects living in an enclosed environment from the evolution of OXPHOS genes.

## Figures and Tables

**Table 1:** Positively selected genes and sites detected in branch-site model tests.

Gene	lnL (Alter)	lnL (Null)	2ΔlnL	*p* Value	Positive Sites
Ndufb5	−5095.22	−5100.57	10.69	0.001	75 G **
Ndufb10	−3997.50	−4001.73	8.45	0.004	118 N **
COX11	−4689.52	−4691.67	4.28	0.039	92 I *, 171 M **
ATPd	−4605.99	−4608.06	4.13	0.042	47 S *, 129 Q *
ATPOSCP	−4263.97	−4266.42	4.89	0.027	92 G **, 93 T **

*, posterior probability > 95% and **, posterior probability > 99%. Only the sites with the significant likelihood ratio test and posterior probability are shown and other sites are detailed in supplementary Table S7.

## Supplementary Materials

The following are available online at https://www.mdpi.com/2073-4425/11/11/1353/s1: Figure S1: The phylogenetic tree of COX4 genes; Table S1: List of fig wasp species used in this study; Table S2: The number of nuclear OXPHOS genes of each species in fig wasps; Table S3: The amino acid substitution rate estimation based on concatenated genes; Table S4: The details of the amino acid substitution rate estimation of each OXPHOS-related gene; Table S5: The one-ratio model results of mitochondrial and nuclear OXPHOS genes; Table S6: The free-ratio model results of mitochondrial and nuclear OXPHOS genes; Table S7: The branch-site model results of mitochondrial and nuclear OXPHOS genes.
