# Supplementary material for: Evolution of Oxidative Phosphorylation (OXPHOS) Genes Reflecting the Evolutionary and Life Histories of Fig Wasps (Hymenoptera, Chalcidoidea)
Source: Genes (Basel). 2020 Nov 15;11(11):1353. doi: 10.3390/genes11111353 (PMC7697784; doi:10.3390/genes11111353)
Supplement: Supplementary file 1 [file genes-11-01353-s001.zip › Figure S1.docx]

**
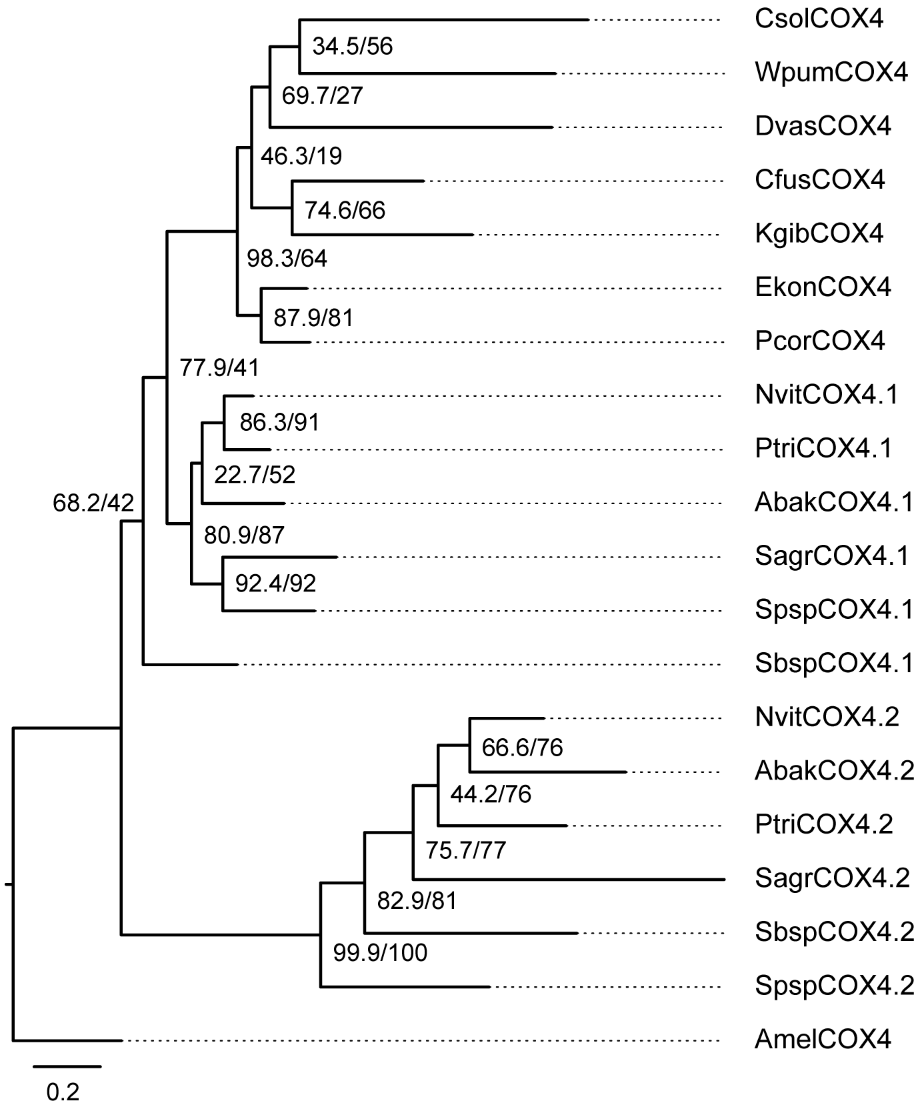
**

**Figure S1.** The phylogenetic tree of COX4 genes. The protein sequences of 12 fig wasps (11 fig wasps mentioned in the main text and pollinator *Ceratosolen solmsi*), *Apis mellifera* (as the outgroup) and *Nasonia vitripennis* (the related species) were aligned using MAFFT v7.313. The tree was reconstructed using IQ-TREE v1.6.1 with 5,000 bootstrap replications. Number on the node represents SH-aLRT%/UFBoot%. Branch lengths represent the average number of amino acid substitutions per site. The COX4 genes of *C. solmsi* are abbreviated to CsolCOX4. The COX4 genes of *A. mellifera* are abbreviated to AmelCOX4. The COX4 genes of *N. vitripennis* are abbreviated to NvitCOX4. Other fig wasp species abbreviations can be found in Table S1.
