# Supplementary material for: Evolution of Oxidative Phosphorylation (OXPHOS) Genes Reflecting the Evolutionary and Life Histories of Fig Wasps (Hymenoptera, Chalcidoidea)
Source: Genes (Basel). 2020 Nov 15;11(11):1353. doi: 10.3390/genes11111353 (PMC7697784; doi:10.3390/genes11111353)
Supplement: Supplementary file 1 [file genes-11-01353-s001.zip › Table S1.docx]

**Table S1.** List of fig wasp species used in this study.

| Group | Mitochondrial genome accession | Nuclear genome accession | Species | Symbol | Subfamily | Family |
| --- | --- | --- | --- | --- | --- | --- |
| Pollinators | MT947596 | JACCHY000000000 | *Dolichoris vasculosae* | Dvas | Agaoninae | Agaonidae |
|  | MT947601 | JACCHZ000000000 | *Wiebesia pumilae* | Wpum | Agaoninae | Agaonidae |
|  | MT947597 | JACCHV000000000 | *Eupristina koningsbergeri* | Ekon | Agaoninae | Agaonidae |
|  | MT947604 | JACCHW000000000 | *Platyscapa corneri* | Pcor | Agaoninae | Agaonidae |
|  | MT916179 | RCIC00000000 | *Ceratosolen fusciceps* | Cfus | Kradibiinae | Agaonidae |
|  | MT947598 | JACCHX000000000 | *Kradibia gibbosae* | Kgib | Kradibiinae | Agaonidae |
| Non-pollinators | MT947599 | JACCIE000000000 | *Sycophaga agraensis* | Sagr | Sycophaginae | Agaonidae |
|  | MT947600 | JACCIC000000000 | *Sycobia* sp.2 | Sbsp | Epichrysomallinae | Pteromalidae |
|  | MT906648 | JACCIA000000000 | *Apocrypta bakeri* | Abak | Sycoryctinae | Pteromalidae |
|  | MT947602 | JACCIB000000000 | *Philotrypesis tridentata* | Ptri | Sycoryctinae | Pteromalidae |
|  | MT947603 | JACCID000000000 | *Sycophila* sp.2 | Spsp | - | Eurytomidae |

Species classification was based on the website (<http://www.figweb.org/Fig_wasps/Classification/index.htm>).
