# Supplementary material for: Evolution of Oxidative Phosphorylation (OXPHOS) Genes Reflecting the Evolutionary and Life Histories of Fig Wasps (Hymenoptera, Chalcidoidea)
Source: Genes (Basel). 2020 Nov 15;11(11):1353. doi: 10.3390/genes11111353 (PMC7697784; doi:10.3390/genes11111353)
Supplement: Supplementary file 1 [file genes-11-01353-s001.zip › Table S2.docx]

**Table S2.** The number of nuclear OXPHOS genes of each species in fig wasps.

| Serial number | Gene | Complex | Pollinators | | | | | |  | Non-pollinators | | | | |
| --- | --- | --- | --- | --- | --- | --- | --- | --- | --- | --- | --- | --- | --- | --- |
|  |  |  | Dvas | Wpum | Ekon | Pcor | Cfus | Kgib |  | Sbsp | Abak | Ptri | Sagr | Spsp |
| 1 | Ndufa2 | I | 1 | 1 | 1 | 1 | 1 | 1 |  | 1 | 1 | 1 | 1 | 1 |
| 2 | Ndufa6 | I | 1 | 1 | 1 | 1 | 1 | 1 |  | 1 | 1 | 1 | 1 | 1 |
| 3 | Ndufa9 | I | 1 | 1 | 1 | 1 | 1 | 1 |  | 1 | 1 | 1 | 1 | 1 |
| 4 | Ndufb10 | I | 1 | 1 | 1 | 1 | 1 | 1 |  | 1 | 1 | 1 | 1 | 1 |
| 5 | Ndufb2 | I | 1 | 1 | 1 | 1 | 1 | 1 |  | 1 | 1 | 1 | 1 | 1 |
| 6 | Ndufb5 | I | 1 | 1 | 1 | 1 | 1 | 1 |  | 1 | 1 | 1 | 1 | 1 |
| 7 | Ndufb7 | I | 1 | 1 | 1 | 1 | 1 | 1 |  | 1 | 1 | 1 | 1 | 1 |
| 8 | Ndufs4 | I | 1 | 1 | 1 | 1 | 1 | 1 |  | 1 | 1 | 1 | 1 | 1 |
| 9 | Ndufs6 | I | 1 | 1 | 1 | 1 | 1 | 1 |  | 1 | 1 | 1 | 1 | 1 |
| 10 | Ndufs8 | I | 1 | 1 | 1 | 1 | 1 | 1 |  | 1 | 1 | 1 | 1 | 1 |
| 11 | Ndufv1 | I | 1 | 1 | 1 | 1 | 1 | 1 |  | 1 | 1 | 1 | 1 | 1 |
| 12 | SDHD | II | 1 | 1 | 1 | 1 | 1 | 1 |  | 1 | 1 | 1 | 1 | 1 |
| 13 | QCR10 | III | 1 | 1 | 1 | 1 | 1 | 1 |  | 1 | 1 | 1 | 1 | 1 |
| 14 | QCR6 | III | 1 | 1 | 1 | 1 | 1 | 1 |  | 1 | 1 | 1 | 1 | 1 |
| 15 | QCR8 | III | 1 | 1 | 1 | 1 | 1 | 1 |  | 1 | 1 | 1 | 1 | 1 |
| 16 | COX10 | IV | 1 | 1 | 1 | 1 | 1 | 1 |  | 1 | 1 | 1 | 1 | 1 |
| 17 | COX11 | IV | S | 1 | 1 | 1 | 1 | 1 |  | 1 | 1 | 1 | 1 | 1 |
| 18 | COX17 | IV | 1 | 1 | 1 | 1 | 1 | 1 |  | 1 | 1 | 1 | 1 | 1 |
| 19 | COX5A | IV | 1 | 1 | 1 | 1 | 1 | 1 |  | 1 | 1 | 1 | 1 | 1 |
| 20 | ATPalpha | V | 1 | 1 | 1 | 1 | 1 | 1 |  | 1 | 1 | 1 | 1 | 1 |
| 21 | ATPb | V | 1 | 1 | 1 | 1 | 1 | 1 |  | 1 | 1 | 1 | 1 | 1 |
| 22 | ATPbeta | V | 1 | 1 | 1 | 1 | 1 | 1 |  | 1 | 1 | 1 | 1 | 1 |
| 23 | ATPc | V | 1 | 1 | 1 | 1 | 1 | 1 |  | 1 | 1 | 1 | 1 | 1 |
| 24 | ATPd | V | 1 | 1 | 1 | 1 | 1 | 1 |  | 1 | 1 | 1 | 1 | 1 |
| 25 | ATPe | V | 1 | 1 | 1 | 1 | 1 | S |  | S | 1 | 1 | 1 | S |
| 26 | ATPepsilon | V | 1 | 1 | 1 | 1 | 1 | 1 |  | 1 | 1 | 1 | 1 | 1 |
| 27 | ATPf | V | 1 | 1 | 1 | 1 | 1 | 1 |  | 1 | 1 | 1 | 1 | 1 |
| 28 | ATPg | V | 1 | 1 | 1 | 1 | 1 | 1 |  | 1 | 1 | 1 | 1 | 1 |
| 29 | ATPgamma | V | 1 | 1 | 1 | 1 | 1 | 1 |  | 1 | 1 | 1 | 1 | 1 |
| 30 | ATPOSCP | V | 1 | 1 | 1 | 1 | 1 | 1 |  | 1 | 1 | 1 | 1 | 1 |
| 31 | Ndufa13 | I | 1 | 2 | 1 | 1 | 1 | 1 |  | 1 | 1 | 1 | 1 | 1 |
| 32 | Ndufb3 | I | 1 | 1 | 1 | 1 | 1 | 1 |  | 2 | 1 | 1 | 1 | 1 |
| 33 | Ndufb6 | I | 1 | 1 | 1 | 1 | 1 | 1 |  | 1 | 1 | 1 | 2 | 1 |
| 34 | Ndufb9 | I | 1 | 1 | 1 | 1 | 1 | 1 |  | 1 | 1 | 1 | 2 | 1 |
| 35 | Ndufs3 | I | 1 | 1 | 2 | 1 | 1 | 1 |  | 1 | 1 | 1 | 1 | 1 |
| 36 | Ndufv2 | I | 1 | 1 | 2 | 1 | 1 | 1 |  | 1 | 1 | 1 | 1 | 1 |
| 37 | SDHC | II | 1 | 1 | 1 | 1 | 1 | 1 |  | 2 | 1 | 2 | 1 | 1 |
| 38 | QCR9 | III | 1 | 1 | 1 | 1 | 1 | 1 |  | 2 | 1 | 1 | 1 | 1 |
| 39 | Cyt1 | III | 1 | 1 | 1 | 1 | 1 | 1 |  | 2 | 2 | 1 | 1 | 1 |
| 40 | QCR2 | III | 2 | 2 | 2 | 2 | 2 | 2 |  | 2 | 2 | 2 | 2 | 2 |
| 41 | COX4 | IV | 1 | 1 | 1 | 1 | 1 | 1 |  | 2 | 2 | 2 | 2 | 2 |
| 42 | COX5B | IV | 1 | 1 | 1 | 1 | 1 | 1 |  | 1 | 1 | 1 | 2 | 1 |
| 43 | ATPdelta | V | 1 | 1 | 2 | 1 | 1 | 1 |  | 1 | 1 | 1 | 1 | 1 |
| 44 | Ndufa10 | I | 1 | 1 | 1 | 1 | 1 | 1 |  | 1 | 1 | 1 | 1 | 1+F |
| 45 | Ndufa11 | I | S+N | 1+N | 1+N | 1+N | S+N | S+N |  | 1+F | 1+S | 1+N | 1+N | 2 |
| 46 | Ndufa12 | I | 1+F | 1+F | 1+S+F | 1+F | 1+F | 1+F |  | 1+F | 1+F | 1+F | 1+S | 2 |
| 47 | Ndufa1 | I | 1 | 0 | 1 | 0 | 0 | 1 |  | 0 | 1 | 1 | 1 | 1 |
| 48 | Ndufa4 | IV | N | 1+F | 1 | 1 | 1 | 1 |  | 1 | S | S | S | 1 |
| 49 | Ndufa5 | I | 1 | 1 | 1 | 1 | 1 | 1 |  | 1 | 1 | F+F | 2 | 1 |
| 50 | Ndufa7 | I | F | F | 1 | F | F | 1 |  | 1 | 1 | F | 1 | 1 |
| 51 | Ndufa8 | I | N | N | N | N | N | N |  | N | N | N | 1 | N+N |
| 52 | Ndufab1 | I | 1 | 1 | 1 | 1 | 1 | 1 |  | 1 | 1 | F | 2 | 1 |
| 53 | Ndufb11 | I | 1 | S | 1+F | S | F | 1 |  | 1 | 1 | 1 | 1 | F |
| 54 | Ndufb1 | I | 1 | 1 | F | 1 | F | 1 |  | 1 | 1 | 1 | F | 1 |
| 55 | Ndufb4 | I | 1 | 1 | 1 | 1 | F | 1 |  | 1 | 1 | 1 | 1 | 1 |
| 56 | Ndufb8 | I | 1 | 1 | 1 | N | 1 | 1 |  | 1 | 1 | F+F | 1 | 1 |
| 57 | Ndufc2 | I | F | 1 | 1 | 0 | 1 | 1 |  | 2 | 1 | 1 | 1 | 1 |
| 58 | Ndufs1 | I | 1 | 1 | 0 | 1 | 1 | 1 |  | 1 | 1 | 1+F | 1 | 1 |
| 59 | Ndufs2 | I | 1 | 1 | 0 | 1 | 1 | 1 |  | 2 | 1 | 1 | 2 | 1 |
| 60 | Ndufs5 | I | F | N | F | N | N | F |  | F | F | F | N | N |
| 61 | Ndufs7 | I | 1+F | 1 | 1 | 1 | 1 | 1 |  | 2 | 1 | 1 | 1 | 1 |
| 62 | SDHA | II | 2 | 1+F | 3 | 2 | 1 | 2 |  | 2 | 2 | 2 | 2 | 2 |
| 63 | SDHB | II | 2 | 2 | 2 | 2 | 1 | 2 |  | 2 | 2 | F+F+F | 2 | 2 |
| 64 | ISP | III | 1+M | 1 | 1 | 1 | 1 | 1 |  | 1+M | 1+M | 1 | 1 | 1 |
| 65 | QCR7 | III | 1 | 1 | 1 | 1 | 1 | 1 |  | 2 | 1 | 1 | 2 | 1+F |
| 66 | COX15 | IV | 1 | 1 | 1 | 1 | 1 | 1 |  | 1 | 1 | 1 | 1 | F+F^*^ |
| 67 | COX6A | IV | 1 | 1 | 2 | 2 | 1+F | 1 |  | 2 | 2 | 2 | 1 | 2 |
| 68 | COX6B | IV | 1+F | 2 | 2 | 2 | 1 | 1 |  | 2 | 2 | 2 | 2 | 2 |
| 69 | COX6C | IV | 0 | 1 | 1 | 1 | 2 | 1 |  | F | 2 | 2 | 1 | 1 |
| 70 | COX7A | IV | 0 | 1 | 2 | 1+F | N | 1 |  | 2 | 2 | 2 | 2 | 2 |
| 71 | COX7C | IV | 0 | 1 | 1 | 1 | 2 | 1 |  | 1 | 1+S | 1+N | F | 1 |
| 72 | ATPf6 | V | 1 | 1 | F | F | F | F |  | F | F | 1 | 1 | F |
| 73 | Total number | - | 77 | 79 | 84 | 78 | 77 | 77 |  | 89 | 85 | 88 | 87 | 85 |

Genes with serial number 1-30 have complete conserved domains and single copy in fig wasps. The genes 31-43 have complete conserved domains and multiple copies in some species. The genes 44-72 have incomplete conserved domains, no conserved domains or no manual annotation results in some species. See full name of each species in Table S1. The numbers below each species represent the gene copy with the following situation: 0, no gene found; N, gene without conserved domain; F, gene with incomplete superfamily conserved domain; S, gene with incomplete specific conserved domain; M, the number of domains of this gene is different from that of other species; 1, gene with complete conserved domain. ^*^ The two sequences of COX15 in Spsp had incomplete conserved domains, one of which was incomplete at the N-terminal and the other at the C-terminal, so we speculated that the two sequences might come from one gene which was separated into two fragments for some reasons; the gene SDHB in Ptri had the similar situation.
