# Supplementary material for: Evolution of Oxidative Phosphorylation (OXPHOS) Genes Reflecting the Evolutionary and Life Histories of Fig Wasps (Hymenoptera, Chalcidoidea)
Source: Genes (Basel). 2020 Nov 15;11(11):1353. doi: 10.3390/genes11111353 (PMC7697784; doi:10.3390/genes11111353)
Supplement: Supplementary file 1 [file genes-11-01353-s001.zip › Table S3.docx]

**Table S3.** The amino acid substitution rate estimation based on concatenated genes.

| Species | Species category | Amino acid rate | | |
| --- | --- | --- | --- | --- |
|  |  | Mitochondrial OXPHOS gene | Nuclear OXPHOS gene | Nuclear non-OXPHOS gene |
| Dvas | Pollinator | 2.456 | 0.916 | 0.581 |
| Wpum | Pollinator | 2.414 | 0.844 | 0.567 |
| Ekon | Pollinator | 2.186 | 0.816 | 0.617 |
| Pcor | Pollinator | 2.353 | 0.847 | 0.577 |
| Cfus | Pollinator | 2.270 | 0.910 | 0.626 |
| Kgib | Pollinator | 2.214 | 0.843 | 0.615 |
| Sbsp | Non-pollinator | 2.207 | 0.752 | 0.590 |
| Abak | Non-pollinator | 1.916 | 0.692 | 0.561 |
| Ptri | Non-pollinator | 1.935 | 0.698 | 0.553 |
| Sagr | Non-pollinator | 2.134 | 0.800 | 0.592 |
| Spsp | Non-pollinator | 1.964 | 0.666 | 0.539 |
| *p* value (Wilcoxon rank sum test) ^#^ | - | 0.009 | 0.004 | 0.126 |
| *p* value (pairwise Wilcoxon rank sum test)* |  | 8.5e-06 | 8.5e-06 | 8.5e-06 |

^#^The *p* values were measured the comparison between pollinators and non-pollinators. *The three comparisons (mitochondrial OXPHOS vs. nuclear OXPHOS, mitochondrial OXPHOS vs. nuclear non-OXPHOS, and nuclear OXPHOS vs. nuclear non-OXPHOS) were conducted by pairwise Wilcoxon rank sum test and *p* values were adjusted by holm correction. The three *p* values were 8.5e-06, individually. See full name of each species in Table S1.
